# Automatically quantified follow-up imaging biomarkers predict clinical outcomes after acute ischemic stroke

**DOI:** 10.3389/fneur.2025.1483138

**Published:** 2025-03-19

**Authors:** Sonya Abraham, Davide Carone, Orell Mielke, Mark Heise, Agnieszka Swierczak, Greg Bass, Stephen Gerry, Zoe V. J. Woodhead, Rafael Namias, James Garrard, David Kallmes, Waleed Brinjikji, Daniel Vaclavik, Petr Mikulenka, Patrick Nicholson, John Thornton, Gary A. Ford, George Harston

**Affiliations:** ^1^CSL Behring, King of Prussia, PA, United States; ^2^Brainomix Limited, Oxford, United Kingdom; ^3^Oxford University Hospitals NHS FT, Oxford, United Kingdom; ^4^CSL Behring, Marburg, Germany; ^5^CSL Innovation, Melbourne, VIC, Australia; ^6^Nuffield Department of Orthopaedics, Rheumatology and Musculoskeletal Sciences, Centre for Statistics in Medicine, University of Oxford, Oxford, United Kingdom; ^7^Mayo Clinic, Rochester, MN, United States; ^8^Nemocnice AGEL, Ostrava, Czechia; ^9^Department of Neurology, Third Faculty of Medicine, Charles University and University Hospital Kralovske Vinohrady, Prague, Czechia; ^10^Beaumont Hospital, Dublin, Ireland; ^11^Department of Radiology, Royal College of Surgeons in Ireland, Dublin, Ireland; ^12^Division of Medical Sciences, University of Oxford, Oxford, United Kingdom

**Keywords:** ischemic stroke, imaging, neuroimaging, follow-up studies, thrombectomy, artificial intelligence

## Abstract

**Background:**

Follow-up infarct volume (FIV) is a proposed surrogate endpoint for proof-of-concept clinical studies in acute ischemic stroke (AIS). This study aimed to provide clinical validation of an automated FIV algorithm, demonstrating the association of imaging biomarkers with clinical outcomes to support the use of these imaging endpoints in clinical trials.

**Methods:**

Data were gathered for adult AIS patients undergoing mechanical thrombectomy with follow-up imaging 12–96 h from initial assessment. Non-contrast computed tomography was used to quantify infarct volume. Image processing used the AI-powered software *Brainomix 360 Stroke* (Brainomix Ltd., Oxford, United Kingdom) and Brainomix core lab research software. Measures included total FIV and components–ischemic injury corrected FIV (cFIV), hemorrhagic transformation (HT), anatomical distortion (AD; a marker of edema) and infarct growth (IG). The primary clinical endpoint was modified Rankin Scale (mRS) at 90 days; secondary clinical endpoint was NIH Stroke Scale (NIHSS) score at 24 h.

**Results:**

Of 986 patients, 843 (85.5%; median age 72 years, 56.7% male) had complete data and were included in the study analysis. Median baseline NIHSS score was 17 (IQR: 12–21). Median imaging follow-up time was 24 h (IQR 20–28). Median 24 h NIHSS score was 11 (5–17); 34% of patients had mRS 0–2 at 90 days. Median FIV was 30.2 mL (12.5–120.8 mL). FIV was significantly associated with 90-day mRS (concordance = 0.819, *p* < 0.001) and NIHSS at 24 h (concordance = 0.722, *p* < 0.001). cFIV, HT, AD, and IG were also significantly associated with good clinical outcomes in both 90-day mRS (concordance = 0.702, *p* < 0.001; 0.660, *p* < 0.001; 0.591, *p* = 0.002; and 0.663, *p* < 0.001, respectively) and NIHSS at 24 h (0.774, *p* < 0.001; 0.652, *p* = 0.004 L; 0.694, *p* < 0.001; and 0.716, *p* < 0.001, respectively). In multivariate analysis, FIV remained strongly associated with 90-day mRS. FIV showed a bimodal distribution consistent with success/failure of recanalization during thrombectomy.

**Conclusion:**

Of the algorithm outputs assessed, FIV was most strongly associated with clinical outcomes. Ischemic injury, HT, edema and IG were also independently significantly associated with clinical outcome. This study validates the prognostic significance of automated FIV and its composites as mechanistic endpoints to improve early-stage trials of therapeutics in AIS.

## Introduction

Despite advances in reperfusion therapy, such as thrombolysis and thrombectomy, a substantial proportion of patients suffering from acute stroke go on to live with moderate to severe disability (1). There is a clear unmet need for additional therapies to augment existing interventions to ensure better long-term outcomes for patients.

A challenge for the development of new treatments in stroke is that regulatory authorities have favored long-term clinical outcomes, such as the residual degree of disability at 90 days measured using the modified Rankin Scale (mRS), as primary endpoints. Such outcomes in ischemic stroke are driven not only by the characteristics of the index stroke event but also by patient and external factors. Confounding variables that can impact outcomes include premorbid health status, neuronal reserve and baseline brain health, established treatments, and external events and illnesses. Showing an effect on disability in a clinical trial can be demanding due to the required trial size, length of follow-up and associated cost. When considering the design of trials for the next generation of stroke treatments, early stage, proof-of-concept trials need endpoints that directly capture the efficacy of an intervention to ensure an efficient transition to later-phase studies. This challenge is not exclusive to stroke therapies, and other fields have successfully addressed the issue by using imaging biomarkers to inform primary endpoints (2).

In acute ischemic stroke, follow-up neuroimaging is typically used to monitor for neurological complications such as hemorrhagic transformation (HT) or cerebral edema (3–7). However, imaging endpoints can also be important for understanding treatment efficacy and mechanisms of action, as well as informing the design of late-phase trials from earlier-phase trial results. In smaller, early-phase trials, changes in mRS may not have been a sufficiently sensitive or reproducible measure of efficacy, especially in the context of treatments providing incremental benefits to an ever-improving standard of care. In clinical trials, follow-up imaging (i.e., after treatment) is typically acquired at around 24 h, in line with consensus guidelines (6), and this can be used to assess the extent of ischemic injury (the follow-up or final infarct volume [FIV]), as well as to investigate the presence of hemorrhage and edema, and the extent of infarct growth since the baseline imaging.

Demands for real-time image interpretation to support treatment decisions have driven innovative technologies such as Artificial Intelligence (AI) analysis to become routine in the standard of care in the acute setting but these technologies have not yet been widely used to characterize follow-up imaging. Automated biomarkers may add value at follow-up through objectivity, timeliness and accuracy, as well as cost efficiency compared to manual quantification by a clinician. To establish a biomarker as a surrogate efficacy endpoint, it should have apparent biological plausibility, it must have prognostic value, and there must be an association between changes in endpoint and changes in clinical outcome where an intervention exists (8).

This study describes the clinical validation of automated follow-up imaging biomarkers as candidates for imaging endpoints in clinical trials, capturing not only the total follow-up lesion volume, but also the relative contributions of infarction, HT, and vasogenic edema to overall lesion volume, acknowledging that the next generation of stroke therapeutics may differentially target these drivers of injury. The objectives were to validate the prognostic association of automated quantification of follow-up imaging biomarkers with long- and short-term clinical outcomes; to investigate independent contributions of sub-components of follow-up infarct volume as predictors of clinical outcome; and to investigate how FIV and its components are modified by recanalization success.

## Methods

### Design

This was a retrospective observational study with data collected from hospital networks in the USA and Europe comprising three hospitals in the US (Mayo Clinic Rochester, Mayo Clinic Jacksonville, and Methodist Healthcare, Memphis), one in Ireland (Beaumont Hospital, Dublin) and four in the Czech Republic (Charles University and University Hospital Kralovske Vinohrady, Nemocnice AGEL Ostrava-Vítkovice, Ostrava University Hospital, and České Budějovice Hospital). Imaging and clinical data were extracted from research registries held at these sites according to the study requirements.

### Patients

Patients with acute ischemic stroke were included if they met the following criteria:

- Large vessel occlusion for consideration of mechanical thrombectomy according to local standard of care.
- 18 years of age or older.
- Follow-up non-contrast computed tomography (NCCT) imaging acquired between 12 and 96 h after baseline computed tomography (CT).
- Sufficient clinical and imaging data available to be included in the analyses.

Patients with evidence of primary intracranial hemorrhage on baseline imaging were excluded. Requiring decompressive hemicraniectomy or presence of herniation on follow-up imaging was not an exclusion criterion for this study.

### Clinical endpoints

Two clinical endpoints were used:

- The primary clinical endpoint was mRS at 90 days (mRS 90). A good clinical outcome was defined as mRS 90 of 2 or less.
- The National Institutes of Health Stroke Scale (NIHSS) score, acquired at approximately the same time as follow-up imaging (around 24 h) was the secondary clinical endpoint. This represents the short-term clinical outcome. A good clinical outcome at 24 h was defined as NIHSS of 2 or less. Early neurological recovery (ENR) was defined using change in NIHSS from baseline to 24 h (or time of follow-up imaging); ENR was defined as an improvement in NIHSS of 8 points or greater, or an NIHSS of 2 or less at 24 h.

Treatment success following mechanical thrombectomy was quantified using the thrombosis in cerebral infarction (TICI) score. Successful recanalization was defined as a final TICI score of 2b–3.

### Imaging biomarkers

For all patients, an NCCT was acquired both at baseline (before treatment) and at follow-up (after treatment, 12–96 h after baseline imaging).

Baseline NCCTs were processed automatically using *Brainomix 360 Stroke* software, an FDA-cleared and CE-marked decision support tool that assesses stroke signs on CT scans to generate an acute ischemic volume (AIV) estimate and ASPECTS (Alberta Stroke Program Early CT Score) for each case (9–13). Follow-up NCCTs were processed using Brainomix in-house software. A Convolutional Neural Network (CNN) based algorithm was used to segment the stroke infarct and to quantify the FIV – the volume of tissue found to be irreversibly injured at follow-up. The FIV CNN is based on a High-Resolution Network (HRNet) (14). The main distinguishing feature between this class of CNNs and other CNNs used for semantic segmentation is that convolutions from high to low resolutions happen in parallel rather than in series, and multi-resolution representations are fused at regular intervals within the CNN. The FIV CNN is trained using the Adam optimizer with a learning rate of 10^−4^ using Combo loss (15) which is a combination of both Dice and Cross Entropy loss. The FIV mask is inferred in 2D, slice-wise, axially through the 3D volume, which is then post-processed. The FIV mask is then refined using automated morphological operations such as island removal techniques and prior brain tissue templates. To ensure that the software performed as expected in this dataset, 40 cases were randomly selected, and the accuracy of the software was compared to the ratings of 3 neuroradiologists (see Supplementary Table S2; Supplementary Figure S4).

The blood detection module from *Brainomix 360 Stroke* was used to detect and quantify HT, calculated as the volume of blood detected on follow-up imaging within the parenchyma.

An image registration approach was then used to estimate (16, 17):

- *Corrected FIV (cFIV)*. This measure corrects FIV for anatomical distortion (AD) caused by edema using a non-linear deformation relative to the baseline image. It represents the volume of tissue damage within the FIV that cannot be accounted for by edema.
- *AD*. This measure is calculated as the difference between FIV and the cFIV. AD represents the volume of tissue distortion caused by edema.
- *Relative AD (rAD)* is calculated as the absolute AD divided by the total FIV. It represents the amount of edema as a proportion of the total volume of damaged tissue.

Finally, the amount of *infarct growth (IG)* between baseline and follow-up was calculated by taking the difference between FIV and AIV. IG represents the growth in the damaged area between baseline and follow-up time points.

Illustration and further explanation of these tissue volumes is given in detail in Supplementary information.

### Statistical analysis

As FIV and cFIV volumes did not have a normal distribution, log transformations were used (log FIV and log cFIV) for all univariate and multivariate analyses.

### Univariate association of FIV with clinical outcome

Univariate analyses were conducted to investigate the association of the imaging biomarkers (log FIV, HT, log cFIV, rAD and IG,) with short- and long-term clinical outcomes. The long- and short-term outcome measures (mRS 90 and 24 h NIHSS) were binarized to classify cases as having good or poor outcomes (using thresholds of mRS ≤2 and NIHSS ≤2 to define good outcome). Binomial regression was used to investigate the association between each imaging biomarker with each binarized clinical outcome measure.

### Multivariate prediction of clinical outcome

Multivariate analyses were used to investigate the prognostic value of the imaging biomarkers on short- and long-term clinical outcomes, while accounting for the impact of other relevant clinical and demographic variables. Two separate regression analyses were conducted, one predicting long-term outcome (binarized mRS 90) and one predicting short-term outcome (24 h NIHSS). For each analysis, three nested models were evaluated:

- Model without FIV: with age, gender and presenting NIHSS as predictors.
- Model with total FIV: with age, gender, presenting NIHSS and log FIV as predictors.
- Model with FIV components: with age, gender, presenting NIHSS, rAD, log cFIV and HT.

Multivariate logistic regression was used for the prediction of binarized mRS 90, and model comparison was conducted using chi-squared tests. Multivariate linear regression was used for the prediction of NIHSS, and model comparison was conducted using F tests. Assumptions of linearity and normality of residuals were checked visually. Multicollinearity of predictors was checked using variance inflation factors (VIF). For the logistic models, pseudo-R^2^ was generated using the method described by Tjur (Tjur’s coefficient of discrimination) (18). Aikaike Information Criterion (AIC) was reported for all models, as a goodness-of-fit metric that takes model complexity into account.

To validate whether FIV is a significant predictor of clinical outcome, taking age, gender and NIHSS into account, the model without FIV was compared with the model with FIV.

To explore whether incorporating the FIV components add more prognostic value compared with FIV alone, the model with FIV was compared with the model with FIV components.

### Impact of recanalization on FIV measures

To explore the association of FIV with success of recanalization, an analysis was performed based on dichotomized TICI after mechanical thrombectomy (successful recanalization: 2b to 3; failed recanalization: 0 to 2a). Although not a randomized intervention, this analysis gives an indication of the effect of treatment on FIV. The difference in FIV between patients with successful and failed recanalization was evaluated using a t-test; as a control, this was compared with the difference in AIV (i.e., ischemic volume prior to mechanical thrombectomy) between the same two patient groups.

This analysis was also performed for HT, AD, and IG to explore the impact of recanalization on these biomarkers.

## Results

An overall cohort of 986 cases was considered suitable for inclusion in the study (Supplementary Table S1). Due to the historical nature of the registries, not all data were available for all patients and although patients with missing imaging data were considered in the full data set of 986 patients, those with missing data were not included in the filtered data set. Of the overall cohort, 843 cases with complete data were included in the study dataset. These patients were from six clinical sites as follows: Beaumont Hospital, Ireland (*n* = 115); Ostrava University Hospital, Czech Republic (*n* = 89); České Budějovice Hospital, Czech Republic (*n* = 63); Mayo Clinic Jacksonville, USA (*n* = 113); Mayo Clinic Rochester, USA (*n* = 237); and Methodist Healthcare, Memphis, USA (*n* = 226). The data used in this study were acquired between the 8th of January 2012 and the 23rd of January 2022.

For the 843 included cases, patients were of median age 72 years [range (IQR) 60–80], and 43.3% female (Table 1). Median NIHSS at baseline was 17 [IQR 12–21]. 61.3% (141/230) had received intravenous thrombolysis. Median imaging follow-up time was 24 h [IQR 20–28]. The distribution of NIHSS at presentation and the time between baseline and follow-up imaging for all cases is illustrated in Supplementary Figure S3.

**Table 1:** Patient demographics overall and according to recanalization status (TICI 2b-3 versus TICI 0-2a).

Patients	*n*	All (*N* = 843)	TICI 2b-3 (*N* = 686)	TICI 0-2a (*N* = 152)
Age	Median (IQR)	72 (60–80)	72 (60–80)	70.5 (60–80)
Gender (F)	% (*n*)	43.3 (365/843)	45.3 (311/686)	34.9 (53/152)
Presenting NIHSS	Median (IQR)	17 (12–21)	17 (12–21)	17 (13–21)
Thrombolysis	% (*n*)	61.3 (141/230)	61.0 (119/195)	60 (18/30)
Follow-up time (hours)	Median (IQR)	24.2 (20.4–28.4)	24.2 (20.4–28.8)	24.0 (20.4–27.9)
24 h NIHSS	Median (IQR)	11 (5–17)	9 (4–16)	17 (13–22)
mRS 90	Median (IQR)	3 (2–5)	3 (1–5)	5 (4–6)
ASPECTS	Median (IQR)	9 (7–10)	9 (8–10)	8 (7–9)
AIV (mL)	Median (IQR)	25.1 (13.7–44.3)	24.5 (13.5–43.2)	29.2 (16.3–46.2)
FIV (mL)	Median (IQR)	30.2 (12.5–120.8)	25.5 (10.5–97.8)	109 (24.5–179.2)
IG (mL)	Median (IQR)	24.8 (9.2–102.0)	20.7 (7.2–85.0)	96 (20.2–162.3)
cFIV(mL)	Median (IQR)	17.9 (6.2–83.7)	14.3 (5.5–62.2)	76.8 (12.7–131.1)
AD (mL)	Median (IQR)	11.7 (2.2–32.5)	8.9 (1.6–25.3)	24.4 (9.6–50.2)
rAD (mL)	Median (IQR)	0.3 (0.1–0.5)	0.3 (0.1–0.5)	0.3 (0.2–0.4)
HT (mL)	Median (IQR)	1.2 (0–10.5)	1 (0–9.6)	2.2 (0–11.8)

AD, anatomical distortion; AIV, acute ischemic volume; ASPECTS, Alberta stroke programme early CT score; cFIV, corrected follow-up infarct volume; F, female; FIV, follow-up infarct volume; HT, hemorrhagic transformation; IG, infarct growth; mRS, modified Rankin Scale; NIHSS, National Institutes of Health Stroke Scale; IQR, interquartile range; rAD, relative anatomical distortion; TICI, thrombolysis in cerebral ischemia.

Final TICI scores were known for all but five cases. Good recanalization (TICI 2b-3) was seen for 686 cases and poor recanalization (TICI 0-2a) for 152 cases.

Clinical outcomes are shown in Table 2 which details the proportion of patients achieving good long- (mRS 90) and short-term (24 h NIHSS) clinical outcomes. Better outcomes were seen for patients with good recanalization.

**Table 2:** Clinical endpoints by recanalization status.^a^

Variable, % (*n*/*N*)	Total population (*N* = 843)	Good recanalization, TICI: 2b–3 (*n* = 686)	Poor recanalization, TICI: 0–2a (*n* = 152)
Good long-term clinical outcome, mRS 90: 0–2	34.28 (289/843)	38.78 (266/686)	13.82 (21/152)
Good post-treatment clinical outcome, 24 h NIHSS: 0–2	14.96 (117/782)	17.68 (113/639)	2.82 (4/142)
Early neurological recovery, improvement of NIHSS by 8 points or more	33.25 (260/782)	39.0 (249/639)	7.75 (11/142)

a Recanalization status was not known for five patients.

mRS, modified Rankin Scale; NIHSS, National Institutes of Health Stroke Scale; TICI, thrombolysis in cerebral ischemia.

### Univariate analyses

**Figure 1:**
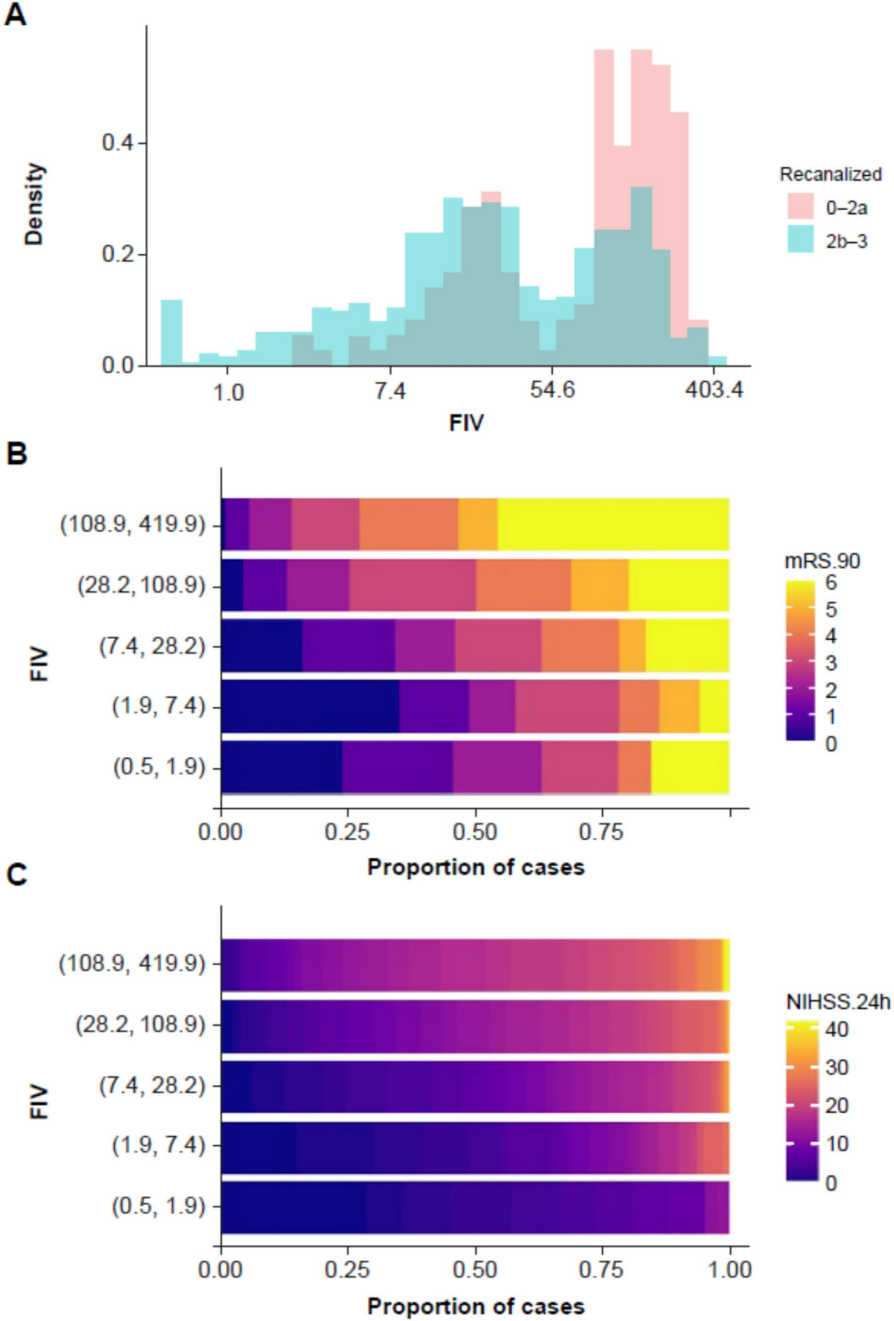
**(A)** Histogram showing distribution of follow-up infarct volume (FIV) as a function of recanalization status, with patients with good recanalization (TICI 2b–3) in blue and patients with poor recanalization (TICI 0–2a) in red. **(B)** Plot showing the association between FIV and mRS at 90 days. Cases were grouped into five bands on the basis of FIV (on the y-axis), and the proportion of patients within each band at each NIHSS score is shown using the color scale along the x-axis. **(C)** The same plot is repeated for the association between FIV and NIHSS at 24 h. FIV, final infarct volume; mRS, modified Rankin Scale; NIHSS, National Institutes of Health Stroke Scale; TICI, thrombolysis in cerebral ischemia.

The distribution of FIV as a function of recanalization status is shown in Figure 1. There was a bimodal distribution of FIV, driven by larger infarcts in patients with poor recanalization (TICI 0–2a). Figure 1 also shows the association between FIV and long-term clinical outcome (mRS 90) as well as short-term clinical outcome (24 h NIHSS): larger infarct volumes were associated with worse clinical outcomes. An ordinal logistic regression showed a significant relationship between log FIV and mRS 90 [odds ratio (OR) =1.71, *t* = 12.24, *p* < 0.001].

A linear regression between FIV and 24 h NIHSS showed a significant relationship [*b* = 2.71, *t*(780) = 16.60, *p* < 0.001; adjusted *R*^2^ = 0.261].

The relationships between the imaging biomarkers and short- and long-term clinical outcomes, dichotomized to good or bad outcome, were confirmed by univariate analyses (binomial regression; Table 3). This showed a significant association between FIV and mRS 90 (OR = 1.668, *p* < 0.001) and NIHSS (OR = 2.142, *p* < 0.001) Significant associations were also seen for all other imaging biomarkers. Out of all biomarkers, FIV showed the strongest concordance with both mRS 90 and 24 h NIHSS.

**Table 3:** Results of the univariate logistic regression analyses, exploring the relationships between follow-up imaging biomarkers and good clinical outcome (a binary measure) in the long term (mRS 90) and short term (24 h NIHSS).

Biomarker	Association with mRS 90 (*N* = 843)			Association with 24 h NIHSS (*N* = 782)		
	Odds ratio (95% CI)	*P*	Concordance	Odds ratio (95% CI)	*P*	Concordance
Log FIV	1.67 (1.50, 1.86)	<0.001	0.722	2.14 (1.85, 2.50)	<0.001	0.819
Log corrected FIV	1.59 (1.44, 1.76)	<0.001	0.702	1.93 (1.68, 2.25)	<0.001	0.774
Infarct growth, mL	1.01 (1.01, 1.01)	<0.001	0.663	1.02 (1.01, 1.02)	<0.001	0.716
Relative anatomical distortion	2.76 (1.46, 5.31)	0.002	0.591	23.51 (8.15, 73.32)	<0.001	0.694
Hemorrhagic transformation, mL	1.04 (1.02, 1.05)	<0.001	0.660	1.02 (1.01, 1.04)	0.004	0.652

CI, confidence interval; FIV, follow-up infarct volume; mRS, modified Rankin Scale; NIHSS, National Institutes of Health Stroke Scale.

### Multivariate analyses

Multivariate analyses were conducted to further explore the association between FIV and clinical outcomes (mRS 90 and 24 h NIHSS). For each outcome measure, three multivariate models were evaluated. The first used clinical predictors only (age, gender, and presenting NIHSS); this was compared with a second model using clinical predictors *and* FIV; and in the third model, the impact of using the separate components of FIV (HT, cFIV, and rAD) was explored. Long-term clinical outcome (mRS 90) was modeled as a binary variable using logistic regression and short-term clinical outcome (24 h NIHSS) was modeled as a continuous variable using linear regression. The VIF values for all models were under 1.5, indicating low correlations between predictors in the models. The results are shown in Table 4 (for mRS 90 and 24 h NIHSS).

**Table 4:** Predictions of clinical outcomes: multivariate logistic regression models (binarized mRS 90) and multivariate linear regression models (24 h NIHSS).

Multivariate logistic regression models predicting clinical outcome (binarized mRS 90)												
**Predictors**	**Model without FIV**					**Model with FIV**			**Model with FIV components**			
	**Log-odds**	**SE**	**Statistic**	***P***	**Log-odds**	**SE**	**Statistic**	***P***	**Log-odds**	**SE**	**Statistic**	***P***
(intercept)	–1.95	0.48	–4.11	<0.001	–4.36	0.60	–7.33	<0.001	–4.56	0.62	–7.34	<0.001
Age (years)	0.03	0.01	5.17	<0.001	0.04	0.01	6.35	<0.001	0.04	0.01	6.15	<0.001
Gender (F)	0.49	0.17	2.94	0.003	0.67	0.18	3.76	<0.001	0.70	0.18	3.88	<0.001
Presenting NIHSS	0.12	0.01	8.64	<0.001	0.10	0.01	6.75	<0.001	0.70	0.02	6.70	<0.001
Recanalization	–1.70	0.27	–6.32	<0.001	–1.31	0.28	–4.65	<0.001	–1.33	0.29	–4.65	<0.001
Log FIV					0.52	0.06	8.16	<0.001				
rAD									1.43	0.39	3.68	<0.001
Log cFIV									0.47	0.07	6.73	<0.001	HT
								0.02	0.01	2.59	0.010	Observations	838				838				838			
R^2^ Tjur	0.187				0.265				0.285				AIC	919				846				828			

Multivariate linear regression models predicting clinical outcome (24 h NIHSS)												
Predictors	Model without FIV				Model with FIV				Model with FIV components			
	Est.	SE	*t*	*P*	Est.	SE	*t*	*P*	Est.	SE	*t*	*P*
(Intercept)	7.31	1.35	5.42	<0.001	−0.68	1.38	−0.50	0.620	−1.71	1.41	−1.21	0.225
Age (years)	−0.01	0.02	−0.65	0.516	0.01	0.01	0.69	0.490	0.01	0.01	0.62	0.537
Gender (F)	0.56	0.49	1.14	0.253	1.08	0.45	2.44	0.0015	1.12	0.44	2.54	0.011
Presenting NIHSS	0.61	0.04	15.64	<0.001	0.49	0.04	13.17	<0.001	0.48	0.04	13.08	<0.001
Recanalization	−6.25	0.62	−10.07	<0.001	−4.35	0.59	−7.41	<0.001	−4.34	0.58	−7.47	<0.001
Log FIV					1.98	0.16	12.76	<0.001				
rAD									6.38	0.97	6.57	<0.001
Log cFIV									1.79	0.16	10.97	<0.001
HT									0.03	0.01	3.03	0.002
Observations	781				781				781			
*R* ^2^	0.312				0.431				0.452			
AIC	5,193				5,046				5,022			

Three models were evaluated in each case. Left: model with clinical variables only, without FIV. Center: model with log FIV as a single predictor. Right: model with FIV components (AD, log cFIV, and HT) as separate predictors. AD, anatomical distortion; AIC, Aikaike information criterion; cFIV, corrected follow-up infarct volume; Est, estimated; F, female;FIV, follow-up infarct volume; HT, hemorrhagic transformation; mRS, modified Rankin Scale; NIHSS, National Institutes of Health Stroke Scale; SE, standard error; rAD, relative anatomical distortion.

The mRS analysis showed lower model fit values than the NIHSS analysis, which was expected given the time difference between the measurement of the predictors and the outcome. However, FIV was seen to be a significant predictor of mRS, and the model with FIV was shown to be a better fit to the data than the model without FIV (*R*^2^ = 0.265 vs. *R*^2^ = 0.187; *p* < 0.001). All three FIV components were shown to be significant predictors of mRS 90, and the overall model fit was significantly better than the model with FIV as a single predictor (*R*^2^ = 0.285 vs. *R*^2^ = 0.265; *p* < 0.001). The AIC for the model with FIV components was the lowest of all three models, indicating that it was the best fit while taking model complexity into account. The multivariate models were repeated using ordinal (rather than binarized) mRS as the outcome variable; similar outcomes were generated but the model fit was improved (Supplementary Table S3).

The NIHSS analysis showed that FIV was a significant predictor of short-term outcome, and that the model with FIV was a significantly better fit to the data than the model without FIV (*R*^2^ = 0.431 vs. *R*^2^ = 0.312; *F* = 162.89, *p* < 0.001). All three FIV components (rAD, cFIV, and HT) were shown to be significant predictors of NIHSS. The model with separate FIV components was shown to be a better fit overall than the model with FIV alone (*R*^2^ = 0.452 vs. *R*^2^ = 0.431; *F* = 14.39, *p* < 0.001). The AIC for the model with FIV components was the lowest of all three models, showing that it was the most parsimonious explanation of the data, even while taking the increased model complexity into account.

### Impact of recanalization on FIV

**Figure 2:**
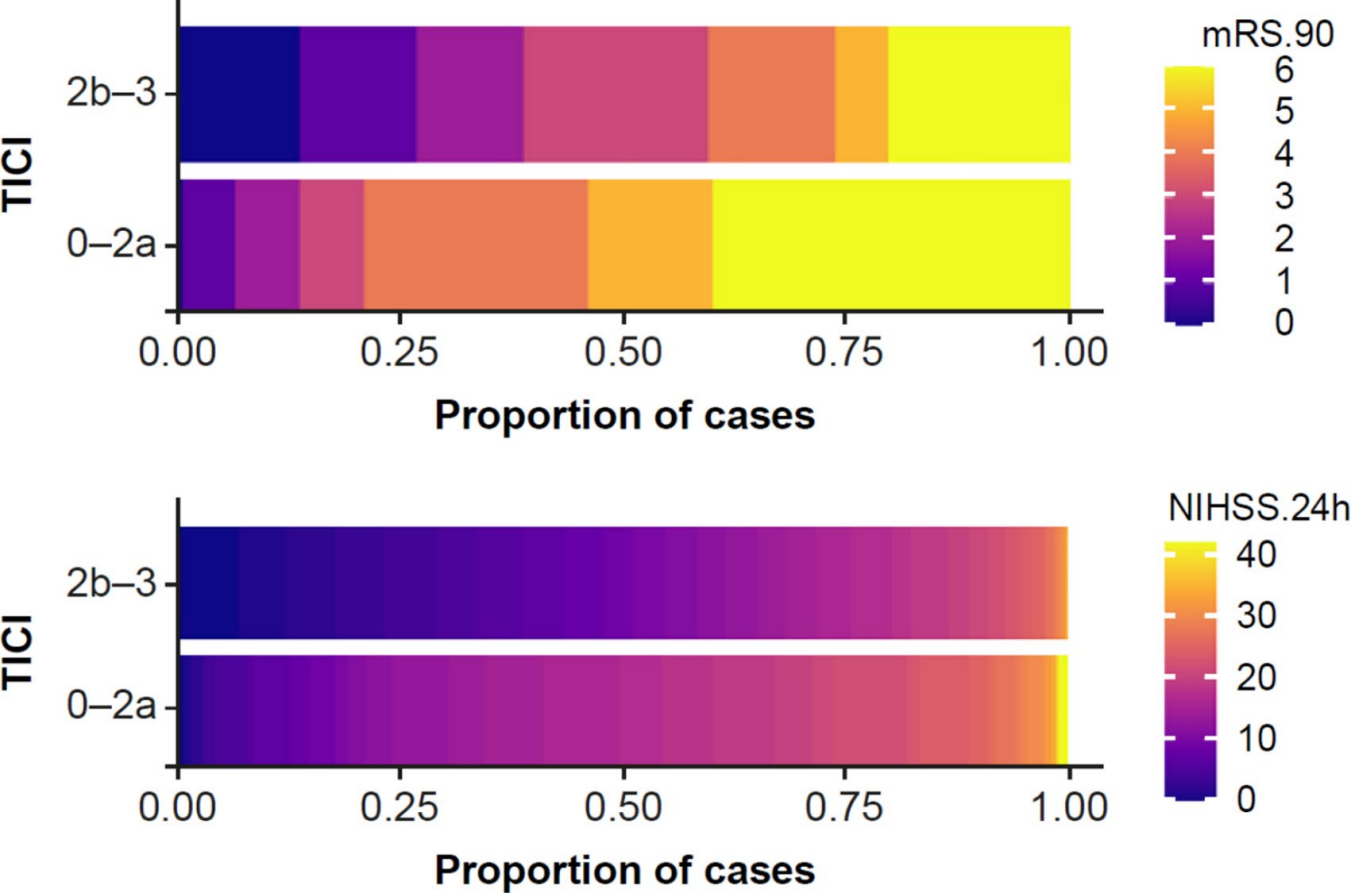
Association between recanalization status (TICI 0–2a versus TICI 2b–3) and (top) long-term clinical outcome, i.e., mRS 90; and (bottom) short-term clinical outcome, i.e., 24 h NIHSS. mRS, modified Rankin Scale; NIHSS, National Institutes of Health Stroke Scale; TICI, thrombolysis in cerebral infarction.

Final TICI score was dichotomized (good recanalization: 2b–3; failed recanalization: 0–2a) and used as a measure of treatment efficacy. The association between recanalization and clinical outcomes (mRS 90 and 24 h NIHSS) were plotted (Figure 2); as expected, this showed better clinical outcomes in patients with good recanalization.

**Figure 3:**
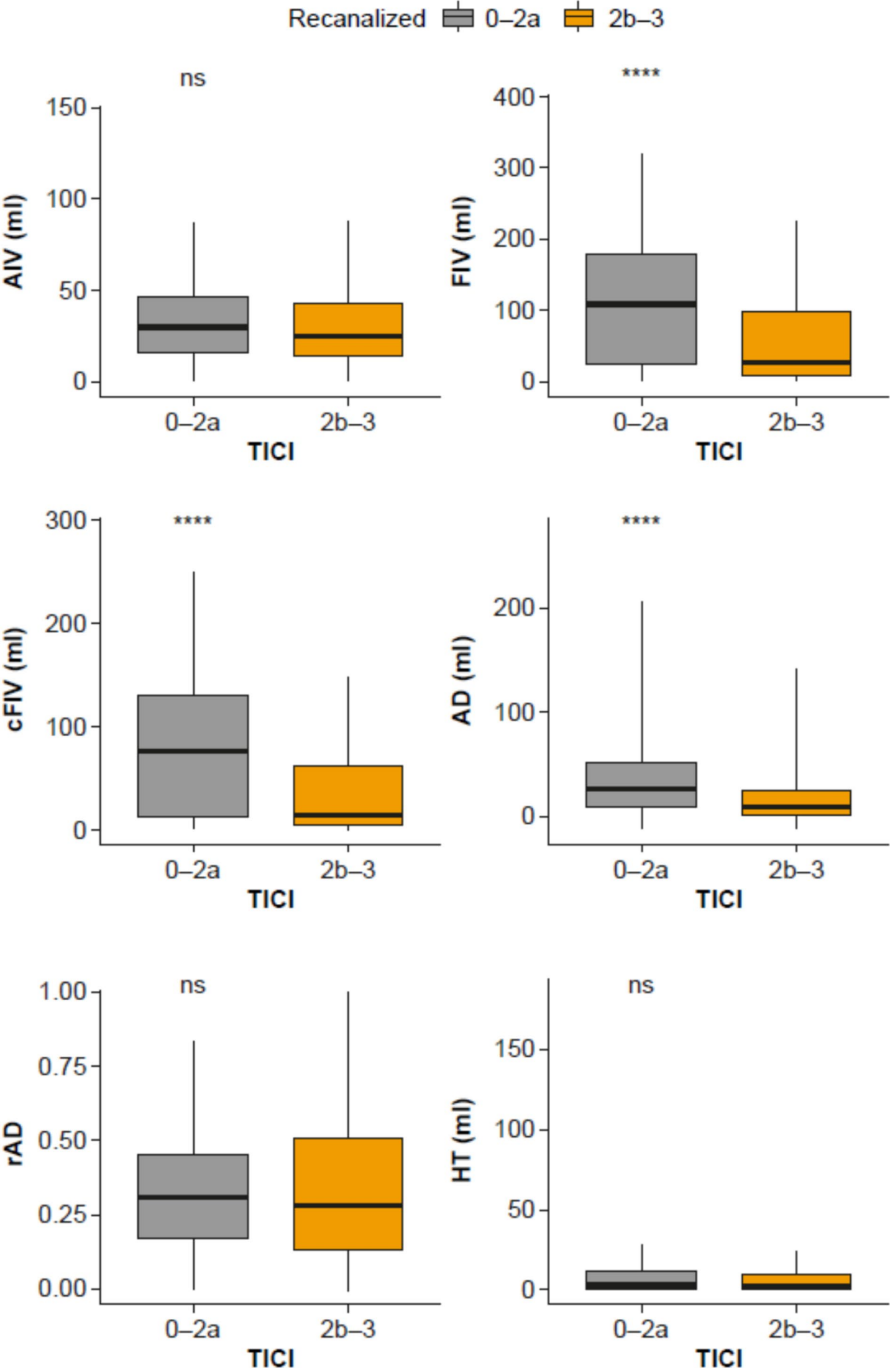
Comparison of biomarkers (AIV, FIV, cFIV AD, rAD and HT) in patients with successful recanalization (orange; TICI 2b–3) versus patients with unsuccessful recanalization (gray; TICI 0–2a). AD, anatomical distortion; AIV, acute ischemic volume; cFIV, corrected FIV; FIV, follow-up infarct volume; HT, hemorrhagic transformation; ns, non-significant; rAD, relative anatomical distortion; TICI, thrombolysis in cerebral infarction. ****p < 0.0001.

In order to explore the impact of treatment on the imaging biomarkers, each biomarker was plotted and compared in the successful and failed recanalization groups using t-tests (Figure 3). A significant difference in FIV was seen between groups, with larger volumes seen in patients with unsuccessful recanalization. As a control, there was no difference between recanalization groups in terms of AIV at baseline, demonstrating that the result seen in FIV was not due to baseline differences between the recanalization groups.

Significant differences between patients with successful and failed recanalization was also seen for cFIV and AD (i.e., more tissue damage that is not accounted for by edema, and greater AD in patients with failed recanalization than those with successful recanalization). No significant differences were seen for rAD or HT.

## Discussion

This study validated the clinical significance of follow-up imaging biomarkers as surrogate endpoints of efficacy for early-stage stroke trials, specifically AI-generated outcomes for FIV and its components: HT, AD caused by edema, and the remaining area of cFIV corrected for edema. The volume of IG was also calculated as the change between the AIV and FIV. A number of these were significantly associated with treatment success and were prognostic for clinical outcomes. Specifically, the biomarkers tested were not only associated with neurological symptoms at the time of image acquisition (24 h NIHSS) but also with long-term disability at 90 days post treatment (mRS 90; primary endpoint). Recanalization success significantly affected FIV, as well as the constituent components of corrected infarct volume, presence of hemorrhage, and the absolute contribution from edema.

Model comparison showed that while FIV was a significant predictor of clinical outcome (taking baseline variables into account), a model with the HT, AD, and cFIV biomarkers as separate predictors was a better fit to the data than a model with FIV alone. This showed that the sub-components of FIV provide additional prognostic value compared with total FIV, in addition to providing insights into mechanism of injury.

In line with previous work, results showed that recanalization had a significant impact on total FIV, as well as cFIV and AD. In contrast, no relationship was observed between recanalization and HT or relative AD, where the volume of AD was expressed as a proportion of total infarct volume (AD divided by FIV). This observation demonstrates that different mechanisms of injury can be differently impacted by treatment effect and may need to be separately captured in trials of neuroprotection. However, importantly for validity of surrogate endpoints, each is also independently associated with prognosis at 90 days.

This study builds on, and is consistent with, previous work. Association between automated measures of AIV and manual or semi-automated measures of FIV has been demonstrated previously (10, 19, 20). AI or machine learning models have been shown to perform at least as well as CT perfusion in predicting FIV (10, 18) and similar can be said for the prognostic value of AIV (from baseline NCCT or CT perfusion) on clinical outcome (21–23). Although a comparison of automated versus manually generated biomarkers was beyond the scope of this study, the former may have advantages in terms of reproducibility, time taken (timely availability of appropriate expertise) and costs; such a comparison is something that may be worthy of future study.

Regarding the predictive value of follow-up imaging biomarkers, it has been shown that FIV (calculated using manual annotation of diffusion-weighted magnetic resonance imaging images) predicts mRS at 90 days, independent of age and baseline stroke severity (24). That study also showed a strong correlation (*r* = 0.98) between FIV and infarct volume at 90 days, validating the use of this endpoint at the 24 h time-point as predictive of longer-term outcomes. The association between manual FIV and mRS 90 has been shown in at least two previous studies (25, 26). This study supports these previous findings, but in contrast used both a fully automated surrogate endpoint, and also a breakdown of the constituent parts of the FIV.

The different components of FIV may have separable clinical impacts and differing responses to experimental treatments. The ability to automatically quantify these components (i.e., measures of edema, HT and ischemic damage) offers the opportunity to investigate the mechanistic effects of treatments in future, using simple imaging that is acquired routinely. In turn, this may inform better patient selection and study design for later-stage clinical trials.

This study has several limitations. It was designed as a retrospective analysis combining datasets from multiple clinical locations; the data collection was not prospective according to a study protocol but reflected the standard of care across different localities. As such, some clinically relevant variables were not known for all cases. Furthermore, retrospective data collection may introduce biases to case selection that would not be the case for prospective or sequential data collection. However, this limitation also points to the generalizability of this approach to multisite retrospective datasets, a common challenge when analyzing imaging from clinical trials. For the analysis exploring the impact of treatment on imaging biomarkers, it should be noted that the use of recanalization as a grouping variable is not fully randomized. There may be systematic differences between the successfully treated and unsuccessfully treated groups that also impacted the biomarkers. Some of these studies have focused on patients with relatively small infarcts which may not be representative of a real-world patient population, and also modeling cannot take into account any post-baseline expansion of infarct which may confound the results. Moreover, the coefficient of determination (R2) for the regression models was low, indicating a relatively high discrepancy between the observed data and the generated fitted values.

In addition, due to AD and rAD estimating the absolute and relative change, respectively, in tissue distortion caused by edema between baseline and follow-up scans, any edema already present in the baseline scan was not included in the AD/rAD calculations. However, considering that one of the inclusion criteria for this study was patient eligibility to undergo thrombectomy, the amount of distortion caused by edema was likely to be minimal. It should be noted that there are no published studies validating the automated quantification of FIV against imaging endpoints; as the focus of the work described here is the validation of this tool on clinical endpoints, such validation was outside the scope of our study. Further work will also be required to validate these findings in prospective randomized controlled trials, where the ability of the biomarkers to capture the impact of a successful treatment can be quantified. In conclusion, this study demonstrates the opportunity for AI-derived imaging biomarkers at follow-up time points to improve the design and delivery of randomized controlled trials in acute ischemic stroke. Imaging biomarkers offer an opportunity to investigate efficacy in early-phase trials and provide insights into the mechanism of action of experimental therapeutics.

## Data Availability

The raw data supporting the conclusions of this article will be made available by the authors, without undue reservation.
