# Supplementary material for: Automatically quantified follow-up imaging biomarkers predict clinical outcomes after acute ischemic stroke
Source: Front Neurol. 2025 Mar 19;16:1483138. doi: 10.3389/fneur.2025.1483138 (PMC11963697; doi:10.3389/fneur.2025.1483138)
Supplement: Supplementary file 1 [file Supplementary_file_1.docx]

# Supplementary Information

## Imaging Biomarkers

Imaging at baseline is routinely used to confirm diagnosis, identify a target for specific treatments, or select patients for clinical trial enrollment based on a predicted capacity to benefit from a specific intervention.[1,2]

Quantification for the extent of brain injury (the ischemic core) at presentation is a common assessment for stroke imaging. This has been achieved using a variety of imaging modalities. Surrogates of perfusion generated in computed tomography (CT), or magnetic resonance imaging (MRI) perfusion studies have been shown to correlate with volumes of tissue at risk of infarction (“the ischemic penumbra”) as well as estimates of ischemic core.[3,4]

A simple model is illustrated in Figure S1.

**Figure S1.** The simple model of brain injury in acute ischemic stroke. These concepts underlying imaging biomarkers at the time of presentation have been used to support the translation of reperfusion therapies into clinical practice.


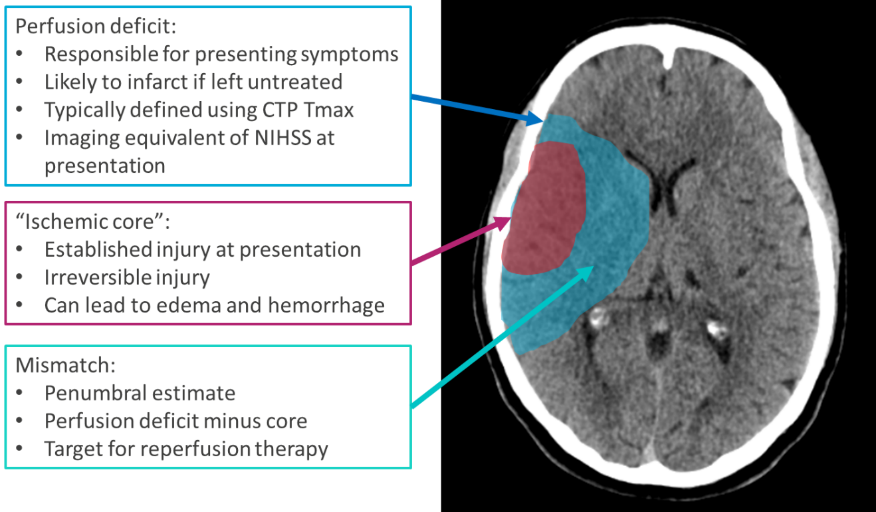


CTP, computed tomography perfusion; NIHSS, National Institutes of Health Stroke Scale; T_max_, time to maximum.

Follow-up imaging is used to evaluate efficacy and safety of an intervention. Repeat imaging is typically acquired on the day following reperfusion therapy administration to investigate the presence of any hemorrhage and/or edema, and to assess the extent of ischemic injury (Figure 2A-C). Consensus guidelines recommend follow-up imaging in clinical trials to occur between 18 and 36h following intervention.[2]

The 24h scan captures the majority of hemorrhagic transformations that occur acutely post stroke.[5] Edema, if present, tends to peak at around day 3 following stroke,[6] but can be reliably identified and quantified at 24h if appropriate methodology is used.[7] Correction of infarct volume for edema can more reliably quantify the true extent of infarction and separate this from the contribution to the volume of abnormal tissue from edema.[8]

The primary biomarker of efficacy that is assessed during follow-up imaging is the follow-up infarct volume (FIV; Figure S2A).[1] Some studies have used follow-up imaging to assess reperfusion or recanalization success, but as this requires contrast administration and is not part of standard of care has not been included in this study. There is some variability in manually segmented FIV lesion masks, even on MRI,[9]. Therefore, Brainomix-developed automated tools were used to extract this from both MRI and CT imaging.

FIV can also be used to produce derivative biomarkers that provide context relative to the presenting imaging such as infarct growth (the additional injury that has accrued between baseline and follow-up) or penumbral salvage (the amount of the tissue at risk that was spared infarction).

Anatomical distortion (AD) captures the contribution of edema and hemorrhagic transformation to the FIV (Figure S2B). AD, in both absolute and relative terms, is associated with clinical outcomes. Absolute AD appears to have a stronger association with clinical outcomes than relative AD (rAD). This is explained by the fact that it is the absolute AD that manifests clinically as a mass effect in the skull and leads to morbidity and mortality. However, because absolute AD is in part driven by the volume of infarcted tissue, using the rAD value allows the impact of edema, or therapeutics driven at this process, to be studied independently from the infarct volume.

The lesser impact of rAD can be attributed to the fact that distortion affecting smaller infarcts will be less significant than that affecting larger volume strokes, even if the rAD value (ie, extent of edema corrected for infarct volume) is the same.

**Figure S2 A–C**: (A) Follow-up infarct volume (FIV) defined on follow-up imaging (CT or MRI), (B) FIV divided into the volume which represents infarcted tissue (corrected FIV [cFIV]), and the volume attributed to edema (anatomical distortion [AD]). For illustration, the FIV is represented by the orange shading. When corrected for AD, the cFIV is denoted by the red dotted line, and the difference is the contribution of AD to the crude FIV. (C) Conventional targets of reperfusion therapy are to minimize infarct growth (the tissue that infarcts between baseline imaging and follow-up) and therefore maximize the percentage of penumbral salvage.

(A)


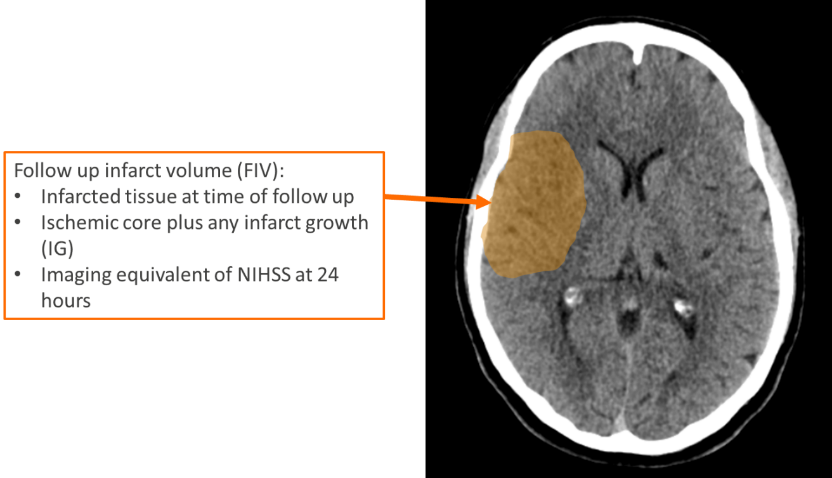


(B)


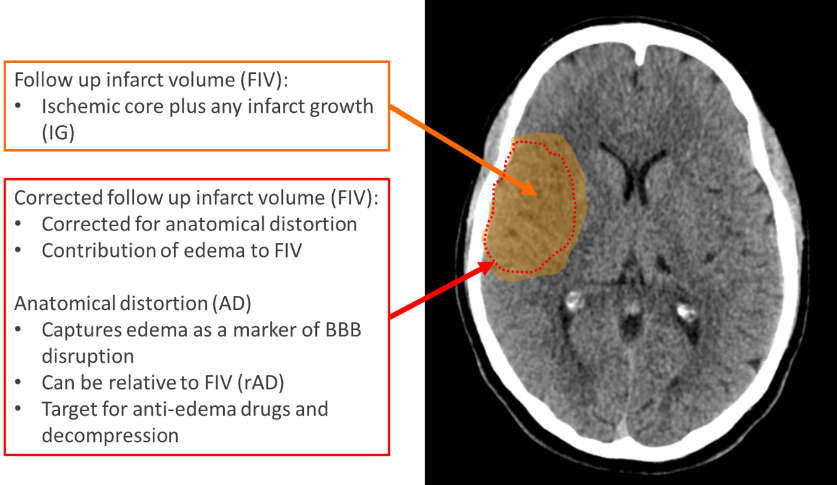


(C)


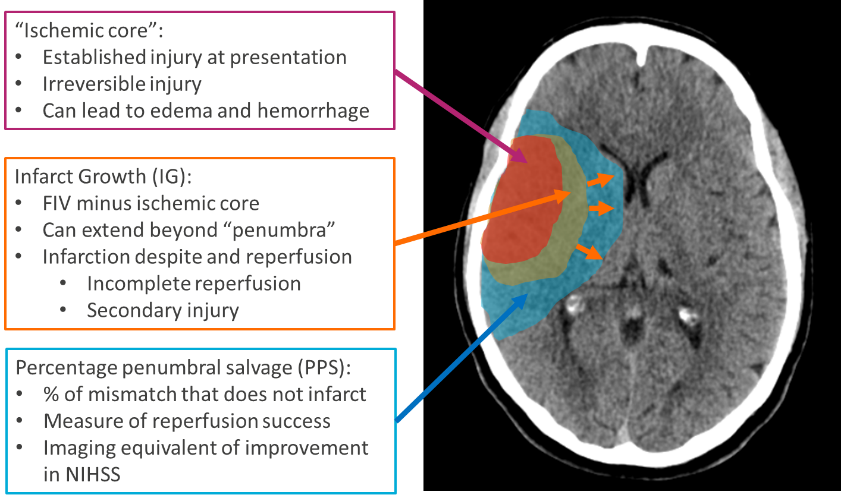


BBB, blood–brain barrier; CT, computed tomography; MRI, magnetic resonance imaging; NIHSS, National Institutes of Health Stroke Scale.

Brainomix software uses artificial intelligence to leverage more information from standard imaging, thereby maximizing the data that can be extracted from diagnostics that are acquired routinely in every hospital. Such imaging biomarkers can also provide greater confidence about the efficacy evaluation of new therapeutics and inform clinical study designs.

Here we have characterized the association of imaging biomarkers with clinical endpoints to evaluate their use as candidate biomarkers in clinical trials.

## Patient Demographics

Due to the historic and variable nature of the registry data, demographic data were not available consistently for all patients. Those data that were available are expressed both in absolute and relative terms below

**Table S1**. Baseline demographics

| Patients | n | 986 |
| --- | --- | --- |
| Age | Median (IQR) | 71 (60–80) |
| Gender (F) | % (n) | 57.4% (566/986) |
| Hypertension | % (n) | 40.9% (110/269) |
| Diabetes | % (n) | 14.1% (38/269) |
| Smoker | % (n) | 6.7% (18/269) |
| Premorbid mRS | Median (IQR) | 0 (0–1) |
| Presenting NIHSS | Median (IQR) | 16 (12–21) |
| Thrombolysis | %(n) | 59.5% (160/269) |
|  |  |  |

CTA, computed tomographic angiography; CTP, computed tomographic perfusion; mRS, modified Rankin Scale; NIHSS, National Institutes of Health Stroke Scale; NCCT, non-contrast computed tomography; MRI, magnetic resonance imaging.

**Figure S3.** Histograms showing distribution of NIHSS at presentation and time of follow-up scan (hours after baseline imaging) for the patient dataset.


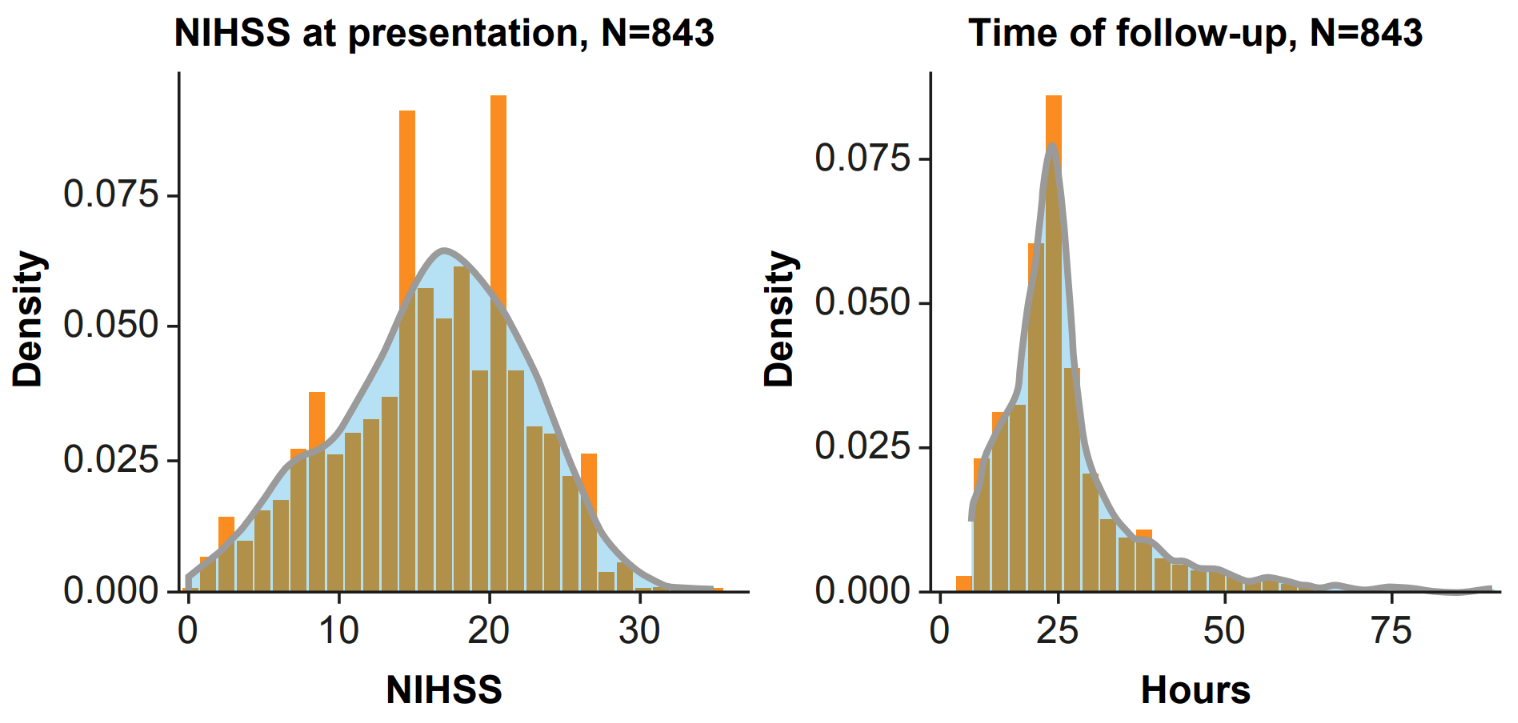


NIHSS, National Institutes of Health Stroke Scale.

## Radiological Variation

To ensure that the software performed as expected in this dataset, 40 cases were randomly selected, and the accuracy of the software was compared to the ratings of 3 neuroradiologists (R1, R2 and R3). Segmentation accuracy was in keeping with expert neuroradiologist (see Table S2 and Figure S4 for a representative case) inter-rater variability, especially in terms of volumetric concordance.

**Table S2.** Dice score, F1 score and volumetric concordance (ICC) between *the Brainomix 360 Stroke* software and each rater, and across raters (R1, R2, and R3) for 40 randomly selected cases.

|  | Mean_Dice | 95% confidence intervals | Mean F1 | 95% Confidence intervals | ICC | 95% Confidence intervals |
| --- | --- | --- | --- | --- | --- | --- |
| FIV_vs_R1 | 0.62 | 0.54–0 .71 | 0.61 | 0.54–0.68 | 0.95 | 0.87–0.98 |
| FIV_vs_R2 | 0.62 | 0.54–0.70 | 0.6 | 0.54–0.67 | 0.97 | 0.95–0.99 |
| FIV_vs_R3 | 0.63 | 0.55–0.71 | 0.61 | 0.55–0.68 | 0.97 | 0.95–0.99 |
| R1_vs_R2 | 0.69 | 0.63–0.76 | 0.66 | 0.60–0.73 | 0.96 | 0.92–0.98 |
| R2_vs_R3 | 0.70 | 0.63–0.78 | 0.67 | 0.60–0.73 | 0.98 | 0.97–0.99 |
| R1_vs_R3 | 0.74 | 0.68–0.79 | 0.64 | 0.56–0.72 | 0.96 | 0.92–0.98 |

FIV, follow up infarct volume; ICC, intraclass correlation coefficient.

**Figure S4.** Example of case in coronal and axial view (A and C). The automated FIV output is outlined in red (B and D) in the same axial slice. The automated FIV output is outlined in red, and example rater segmentations are outlined in green.


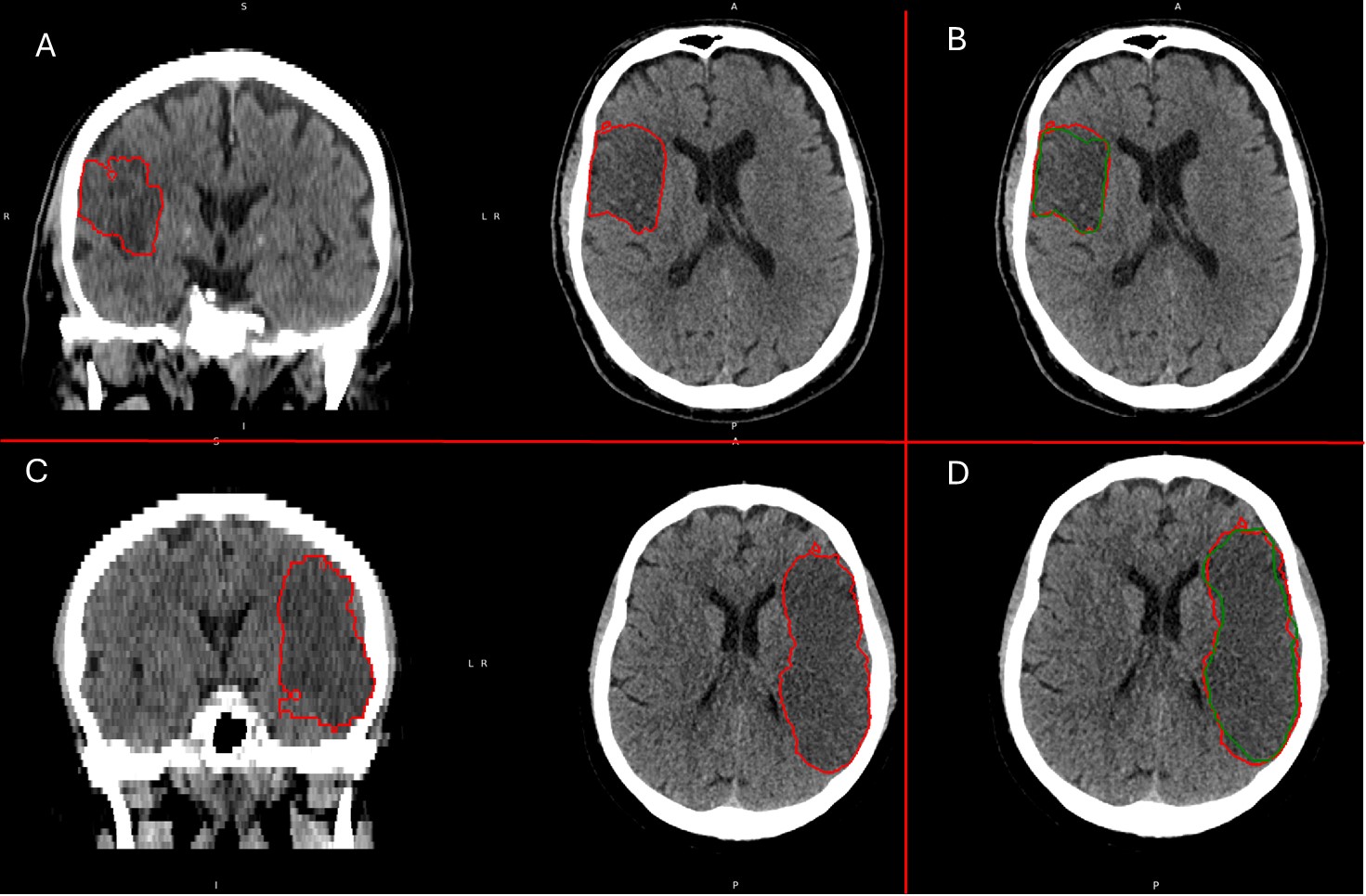


FIV, follow-up infarct volume.

## Additional Multivariate Analysis

**Table S3. Multivariate model analysis using ordinal mRS as the outcome variable**

| **Predictors** | **Model without FIV** | | | | **Model with FIV** | | | | **Model with FIV components** | | | |
| --- | --- | --- | --- | --- | --- | --- | --- | --- | --- | --- | --- | --- |
|  | *Log-Odds* | *Std. error* | *Statistic* | *P value* | *Log-Odds* | *Std. error* | *Statistic* | *P value* | *Log-Odds* | *Std. error* | *Statistic* | *P value* |
| Age | 0.03 | 0.00 | 5.91 | <0.001 | 0.03 | 0.00 | 7.58 | <0.001 | 0.03 | 0.00 | 7.33 | <0.001 |
| Gender (F) | 0.16 | 0.13 | 1.30 | 0.195 | 0.32 | 0.13 | 2.49 | 0.013 | 0.35 | 0.13 | 2.72 | 0.007 |
| NIHSS at baseline | 0.10 | 0.01 | 9.68 | <0.001 | 0.08 | 0.01 | 7.30 | <0.001 | 0.08 | 0.01 | 7.28 | <0.001 |
| Recanalization | −1.43 | 0.17 | −8.65 | <0.001 | −1.06 | 0.17 | −6.25 | <0.001 | −1.12 | 0.17 | −6.52 | <0.001 |
| FIV [log] |  |  |  |  | 0.51 | 0.05 | 10.68 | <0.001 |  |  |  | <0.001 |
| rAD |  |  |  |  |  |  |  |  | 1.50 | 0.29 | 5.13 | <0.001 |
| cFIV [log] |  |  |  |  |  |  |  |  | 0.48 | 0.05 | 9.38 | <0.001 |
| HT |  |  |  |  |  |  |  |  | 0.01 | 0.00 | 3.04 | 0.002 |
| Observations | 838 |  |  |  | 838 |  |  |  | 838 |  |  |  |
| R^2^ Nagelkerke | 0.245 |  |  |  | 0.350 |  |  |  | 0.372 |  |  |  |
| AIC | 2966.727 |  |  |  | 2847.174 |  |  |  | 2822.598 |  |  |  |

AIC, Aikaike information criterion; cFIV, corrected follow-up infarct volume; F, female; FIV, follow-up infarct volume; HT, hemorrhagic transformation; mRS, modified Rankin Scale; NIHSS, National Institutes of Health Stroke Scale; rAD, relative anatomical distortion; Std, standard.

# References

1. Harston GW, Rane N, Shaya G, Thandeswaran S, Cellerini M, Sheerin F, Kennedy J. Imaging biomarkers in acute ischemic stroke trials: A systematic review. *AJNR Am J Neuroradiol* 2015;36(5):839-843.

2. Wintermark M, Albers GW, Broderick JP, Demchuk AM, Fiebach JB, Fiehler J, et al. Acute stroke imaging research roadmap ii. *Stroke* 2013;44(9):2628-2639.

3. Konstas AA, Goldmakher GV, Lee TY, Lev MH. Theoretic basis and technical implementations of ct perfusion in acute ischemic stroke, part 1: Theoretic basis. *AJNR Am J Neuroradiol* 2009;30(4):662-668.

4. Campbell BC, Yassi N, Ma H, Sharma G, Salinas S, Churilov L, et al. Imaging selection in ischemic stroke: Feasibility of automated ct-perfusion analysis. *Int J Stroke* 2015;10(1):51-54.

5. Larrue V, von Kummer R, del Zoppo G, Bluhmki E. Hemorrhagic transformation in acute ischemic stroke. Potential contributing factors in the european cooperative acute stroke study. *Stroke* 1997;28(5):957-960.

6. Hacke W, Schwab S, Horn M, Spranger M, De Georgia M, von Kummer R. 'Malignant' middle cerebral artery territory infarction: Clinical course and prognostic signs. *Archives of neurology* 1996;53(4):309-315.

7. Harston GWJ, Carone D, Sheerin F, Jenkinson M, Kennedy J. Quantifying infarct growth and secondary injury volumes: Comparing multimodal image registration measures. *Stroke* 2018;49(7):1647-1655.

8. Harston GW, Minks D, Sheerin F, Payne SJ, Chappell M, Jezzard P, et al. Optimizing image registration and infarct definition in stroke research. *Ann Clin Transl Neurol* 2017;4(3):166-174.

9. Neumann AB, Jonsdottir KY, Mouridsen K, Hjort N, Gyldensted C, Bizzi A, et al. Interrater agreement for final infarct mri lesion delineation. *Stroke* 2009;40(12):3768-3771.
